# Supplementary material for: Environment Constrains Fitness Advantages of Division of Labor in Microbial Consortia Engineered for Metabolite Push or Pull Interactions
Source: mSystems. 2022 Jun 28;7(4):e00051-22. doi: 10.1128/msystems.00051-22 (PMC9426560; doi:10.1128/msystems.00051-22)
Supplement: FIG S1 [file msystems.00051-22-s0007.pdf]

Supplementary material Figure S1.

A. Single Substrate (Acetate Only, $\mu_m = 0.4\ h^{-1}$)

A1.

Andrews and Noack

$$\mu = \mu_m \left(\frac{1}{1 + \frac{K_A}{A} + \frac{A}{K_I}} \right)$$

$$K_A = 0.072\ mM$$
$$K_I = 0.378\ mM$$
$$R^2 = 0.94$$

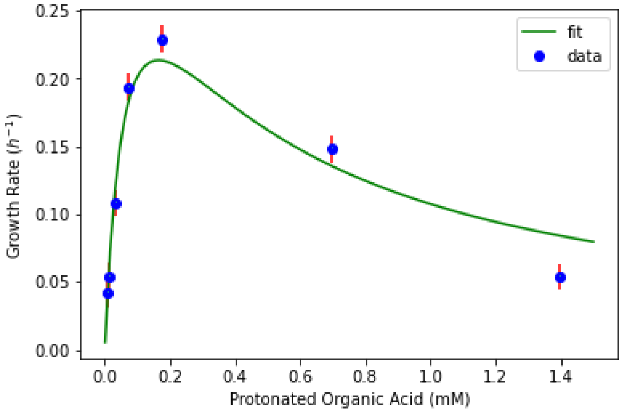

A2.

Webb

$$\mu = \mu_m \left(\frac{A\left(1 + \frac{K_A}{K_I}\right)}{K_A + A + \frac{A^2}{K_I}} \right)$$

$$K_A = 0.128\ mM$$
$$K_I = 0.230\ mM$$
$$R^2 = 0.94$$

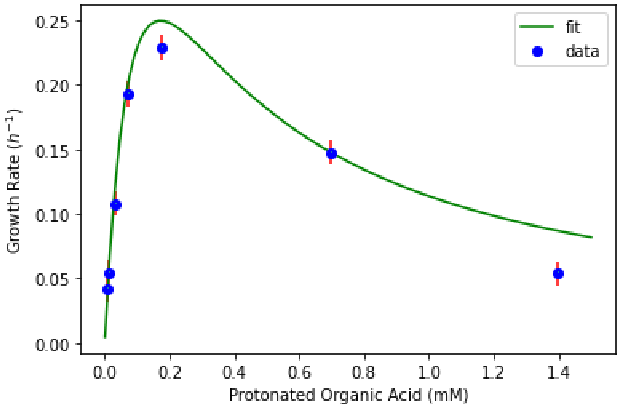

A3.

Yano and co-workers

$$\mu = \mu_m \left(\frac{1}{1 + \frac{K_A}{A} + \frac{A}{K_I} + \left(\frac{A}{K_I}\right)^2} \right)$$

$$K_A = 0.128\ mM$$
$$K_I = 0.230\ mM$$
$$R^2 = 0.98$$

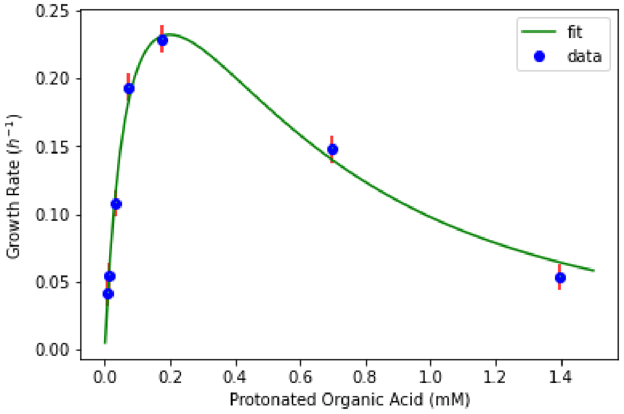

A4.

Aiba and co-workers

$$\mu = \mu_m \left(\frac{A}{K_A + A}\right) e^{-\frac{A}{K_I}}$$

$$K_A = 0.0723\ mM$$
$$K_I = 0.760\ mM$$
$$R^2 = 0.98$$

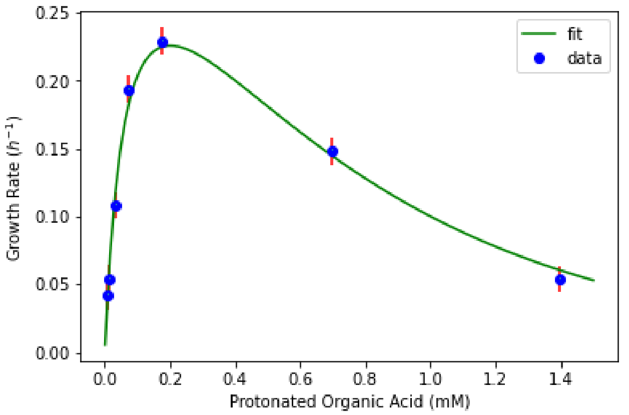

A5.

Tessier-Type

$$\mu = \mu_m \left[e^{-\frac{A}{K_I}} - e^{-\frac{A}{K_A}}\right]$$

$$K_A = 0.0885\ mM$$
$$K_I = 0.672\ mM$$
$$R^2 = 0.97$$

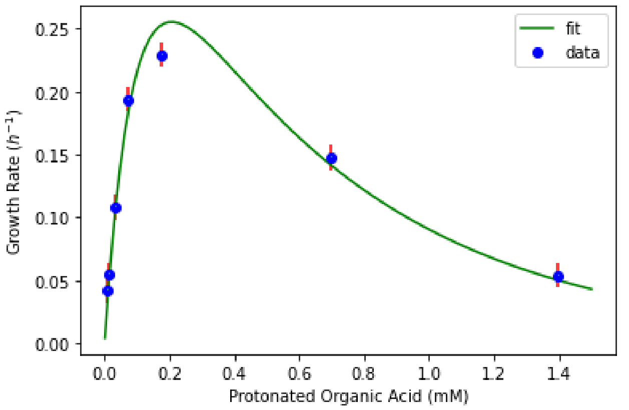

A6.

Webb (with σ)

$$\mu = \mu_m \left(\frac{A}{A + K_A\left(1 + \frac{\sigma}{K_I}\right)}\right) e^{1.17\sigma A}$$

$$K_A = 0.846\ mM$$
$$K_I = 1.23\ mM$$
$$\sigma = -1.12\ mM$$
$$R^2 = 0.98$$

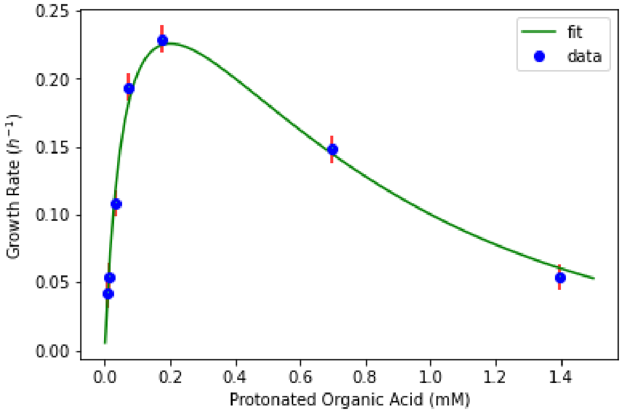

A7.

Wayman and Tseng

$$\mu = \mu_m \left(\frac{A}{K_A + A}\right), A < A'$$

$$\mu = \mu_m \left(\frac{A}{K_A + A}\right) - K_I(A - A'),$$
$$A > A'$$

$A' = 0.139\ mM$
$K_A = 0.124\ mM$
$K_I = 0.659\ mM$
$R^2 = 0.88$

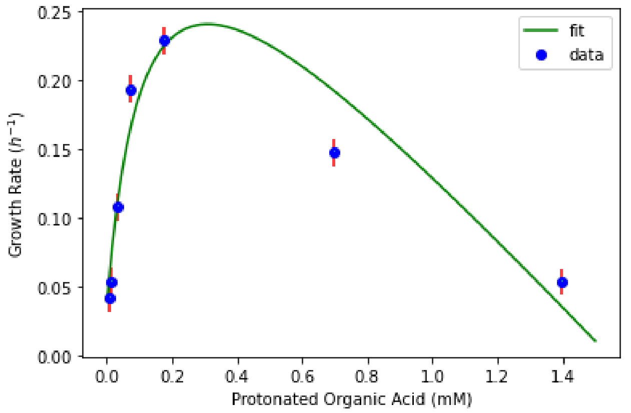

A8.

Classic model

$$\mu = \mu_m \left(\frac{A}{K_A + A}\right)\left(\frac{K_I}{K_I + A}\right)$$

$K_A = 0.0644\ mM$
$K_I = 0.421\ mM$
$R^2 = 0.92$

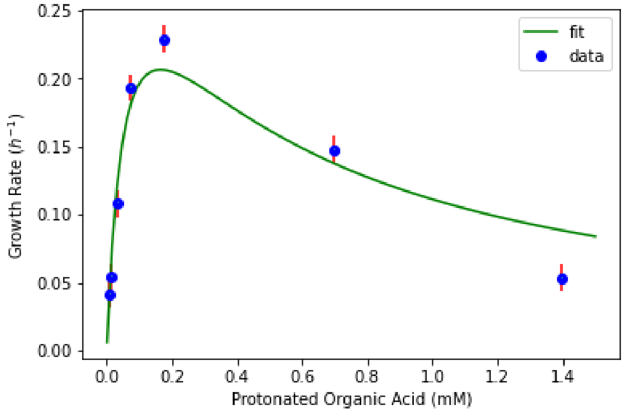

B. Multiple Substrate (Acetate and Glucose, $\mu_m = \mu_G = 0.65\ h^{-1}, \mu_A = 0.4\ h^{-1}, K_G = 0.005\ mM$)

B1.

Dagley and Hinshelwood

$$\mu = \mu_m \left(\frac{G}{K_G + G}\right)(1 - KA)$$

$K = 0.696\ mM^{-1}$
$R^2 = 0.97$

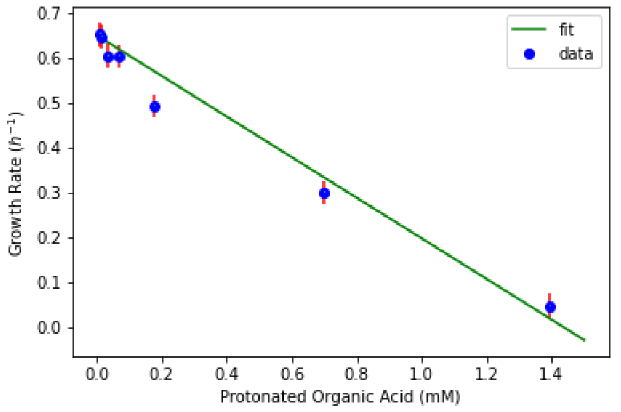

B2.

Holzberg and co-workers

$$\mu = \mu_m - K(A - K_I)$$

$$K \;=\; 0.427\; h^{-1}$$
$$K_I \;=\; -0.060\; mM$$
$$R^2 \;=\; 0.98$$

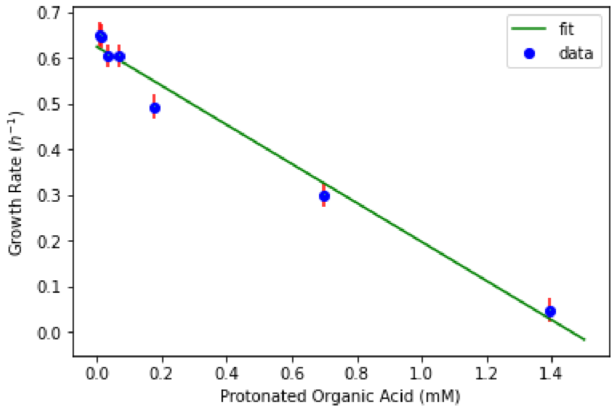

B3.

Ghose and Tyagi

$$\mu = \mu_m \left(1 - \frac{A}{A^*}\right)\left(\frac{G}{K_G + G}\right)$$

$$A^* \;=\; 1.44\; mM$$
$$R^2 \;=\; 0.97$$

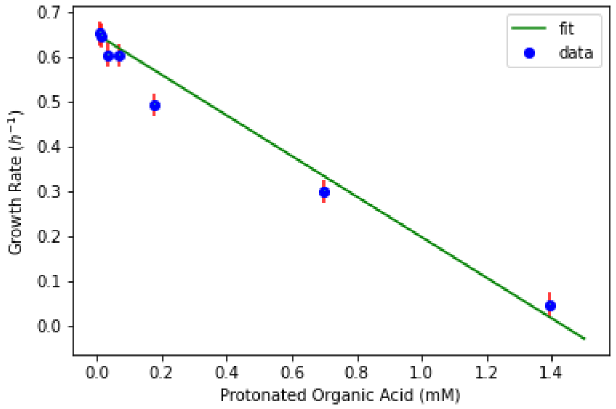

B4.

Aiba and co-workers

$$\mu = \mu_m \left(\frac{G}{K_G + G}\right) e^{-KA}$$

$$K \;=\; 1.356\; mM^{-1}$$
$$R^2 \;=\; 0.98$$

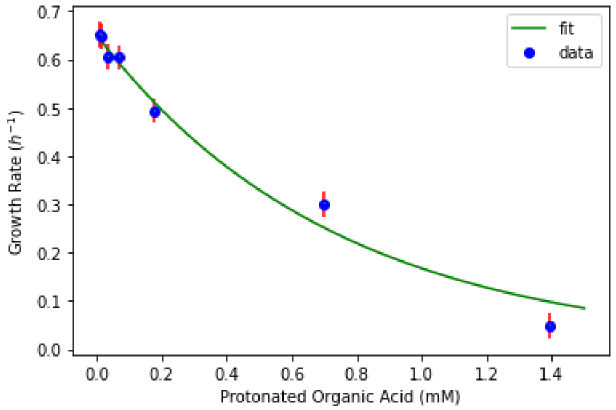

B5.

Jerusalimsky and Neronova

$$\mu = \mu_m \left(\frac{G}{K_G + G}\right)\left(\frac{K_I}{K_I + A}\right)$$

$$K_I \;=\; 0.453\; mM$$
$$R^2 \;=\; 0.95$$

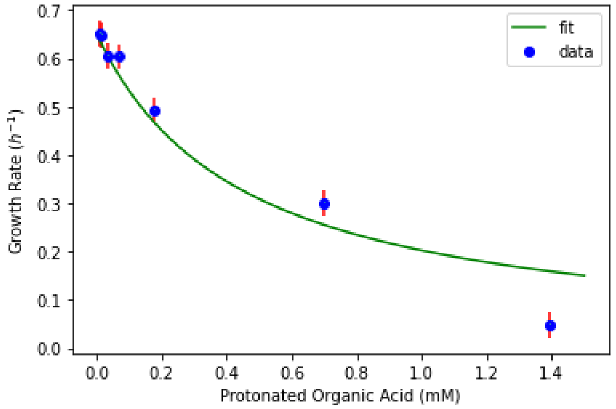

B6.

Aiba and co-workers dual substrate derivative

$$\mu = \left[\mu_G\left(\frac{G}{K_G + G}\right) + \mu_A\left(\frac{A}{K_A + A}\right)\right] e^{-\alpha A}$$

$$K_A = 1.54\ mM$$
$$\alpha = 1.60\ mM^{-1}$$
$$R^2 = 0.98$$

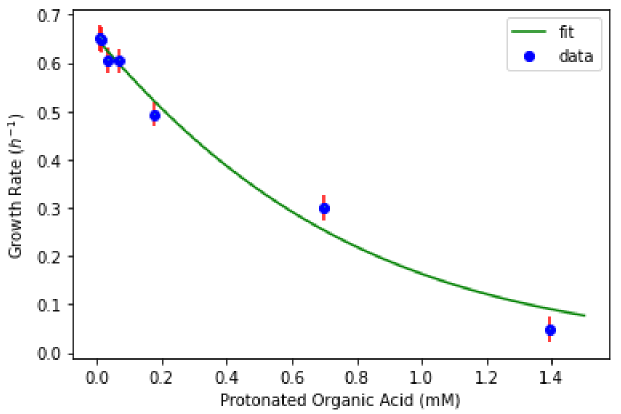

B7.

Aiba and co-workers dual substrate derivative (single μ)

$$\mu = \mu_m\left[\left(\frac{G}{K_G + G}\right) + \left(\frac{A}{K_A + A}\right)\right] e^{-\alpha A}$$

$$K_A = 2.06\ mM$$
$$\alpha = 1.67\ mM^{-1}$$
$$R^2 = 0.98$$

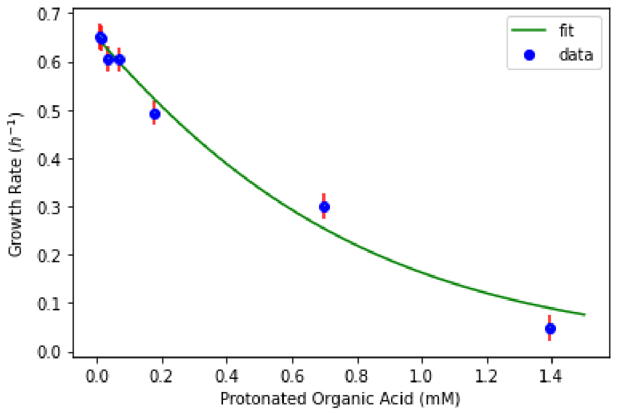

B8.

Classic model dual substrate derivative

$$\mu = \left[\mu_G\left(\frac{G}{K_G + G}\right) + \mu_A\left(\frac{A}{K_A + A}\right)\right]\left(\frac{K_I}{K_I + A}\right)$$

$$K_A = 0.145\ mM$$
$$K_I = 0.224\ mM$$
$$R^2 = 0.96$$

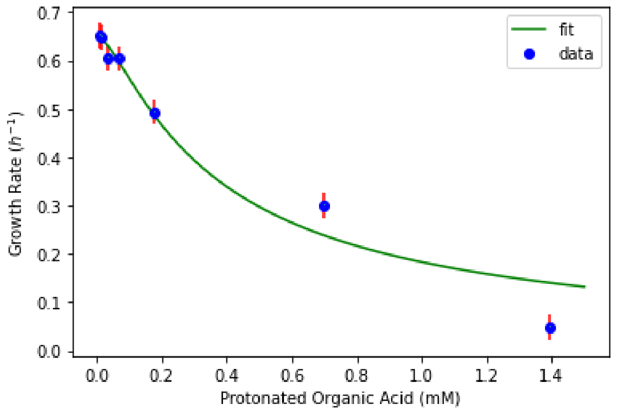

B9.

Classic model dual substrate derivative (single μ)

$$\mu = \mu_m \left[\left(\frac{G}{K_G + G} \right) + \left(\frac{A}{K_A + A} \right) \right] \left(\frac{K_I}{K_I + A} \right)$$

$$K_A = 0.186\ mM$$
$$K_I = 0.180\ mM$$
$$R^2 = 0.96$$

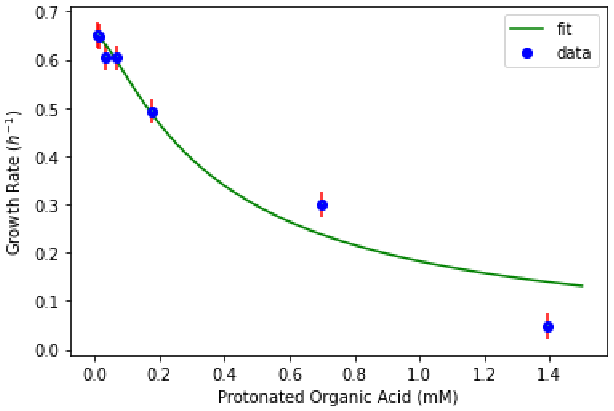

C. Single Substrate (Lactate Only, $\mu_m = 0.5$)

C1.

Andrews and Noack

$$\mu = \mu_m \left(\frac{1}{1 + \frac{K_L}{L} + \frac{L}{K_I}} \right)$$

$$K_L = 0.0037\ mM$$
$$K_I = 0.239\ mM$$
$$R^2 = 0.89$$

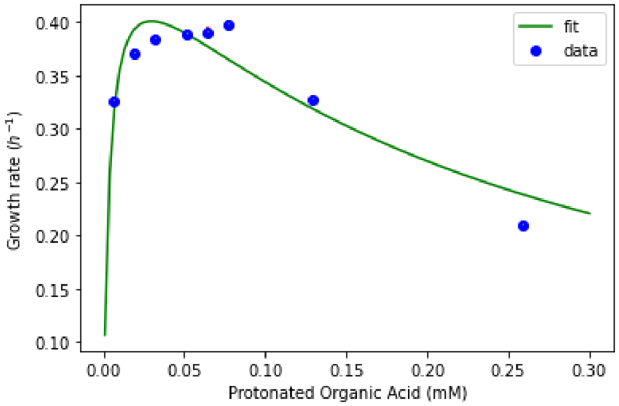

C2.

Webb

$$\mu = \mu_m \left(\frac{L\left(1 + \frac{K_L}{K_I}\right)}{K_L + L + \frac{L^2}{K_I}} \right)$$

$$K_L = 0.00393\ mM$$
$$K_I = 0.227\ mM$$
$$R^2 = 0.89$$

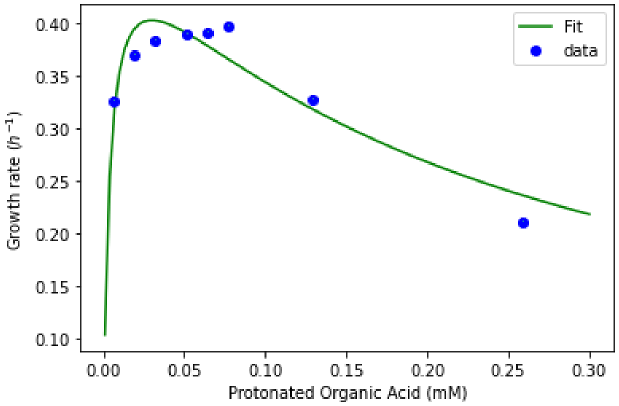

C3.

Yano and co-workers

$$\mu = \mu_m \left(\frac{1}{1 + \frac{K_L}{L} + \frac{L}{K_I} + \left(\frac{L}{K_I}\right)^2} \right)$$

$$K_L = 0.0040\ mM$$
$$K_I = 0.343\ mM$$
$$R^2 = 0.92$$

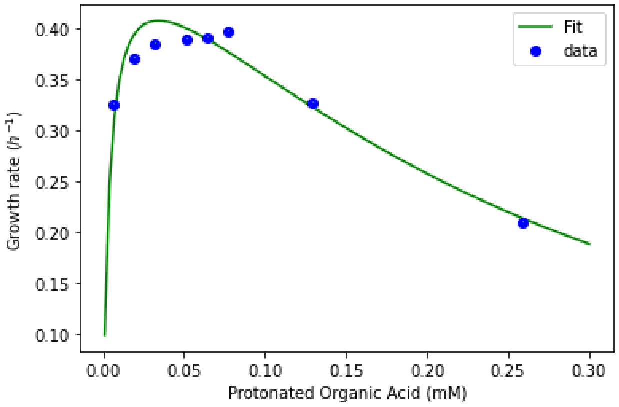

C4.

Aiba and co-workers

$$\mu = \mu_m \left(\frac{L}{K_L + L} \right) e^{-\frac{L}{K_I}}$$

$$K_L = 0.00382\ mM$$
$$K_I = 0.317\ mM$$
$$R^2 = 0.93$$

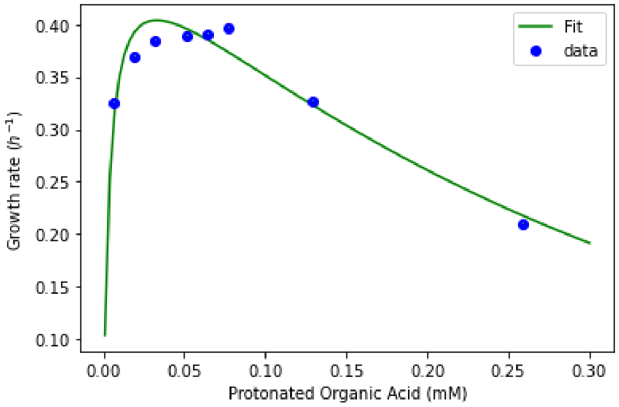

C5.

Tessier-Type

$$\mu = \mu_m \left[e^{-\frac{L}{K_I}} - e^{-\frac{L}{K_L}} \right]$$

$$K_L = 0.00712\ mM$$
$$K_I = 0.275\ mM$$
$$R^2 = 0.64$$

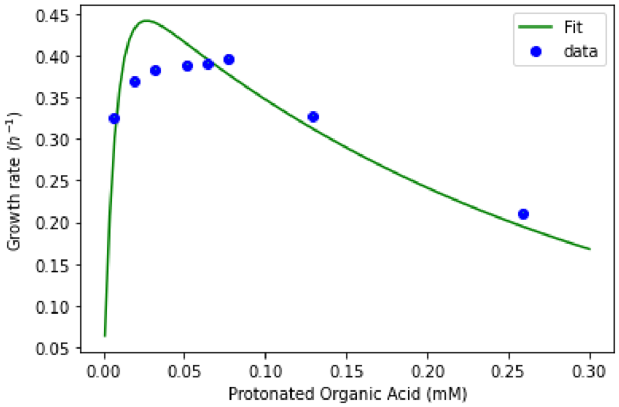

C6.

Webb (with σ)

$$\mu = \mu_m \left(\frac{L}{L + K_L \left(1 + \frac{\sigma}{K_I}\right)} \right) e^{1.17L\sigma}$$

$$K_L = 0.00050\ mM$$
$$K_I = -0.41\ mM$$
$$\sigma = -2.70\ mM$$
$$R^2 = 0.93$$

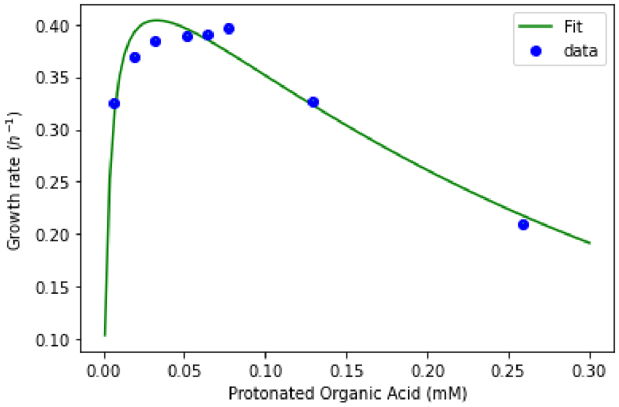

C7.

Wayman and Tseng

$$\mu = \mu_m \left(\frac{L}{K_L + L} \right), L < L'$$

$$\mu = \mu_m \left(\frac{L}{K_L + L} \right) - K_I(L - L'),$$
$$L > L'$$

L' = 0.078 mM

$$K_L = 0.0169\ mM$$
$$K_I = 3.21\ mM$$
$$R^2 = 0.66$$

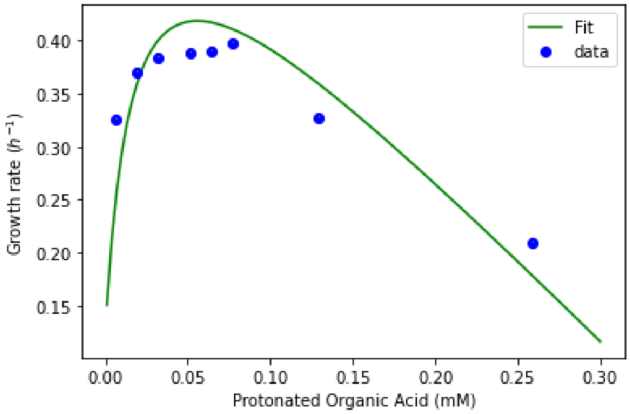

C8.

Classic Model

$$\mu = \mu_m \left(\frac{L}{L + K_L} \right) \left(\frac{K_I}{K_I + L} \right)$$

$$K_L = 0.0035\ mM$$
$$K_I = 0.246\ mM$$
$$R^2 = 0.89$$

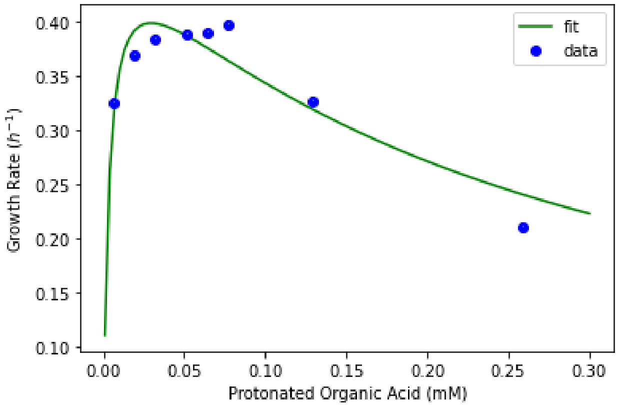

<table>
<tr><th colspan="2">D. Multiple Substrate (Lactate and Glucose, $\boldsymbol{\mu_m = \mu_G = 0.65, \mu_L = 0.5, K_G = 0.005}$)</th></tr>
<tr><td>

D1.

Dagley and Hinshelwood

$$\mu = \mu_m \left(\frac{G}{K_G + G}\right)(1 - KL)$$

$$K = 1.092\ mM$$
$$R^2 = 0.4$$

</td><td>

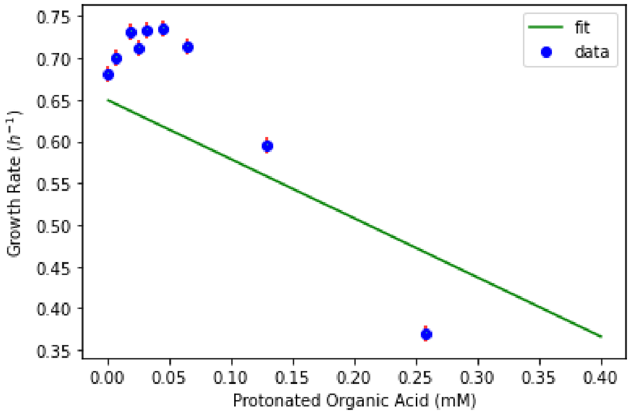

</td></tr>
<tr><td>

D2.

Holzberg and co-workers

$$\mu = \mu_m - K(L - K_I)$$

$$K = 1.347\ mM$$
$$K_I = 0.075\ mM$$
$$R^2 = 0.88$$

</td><td>

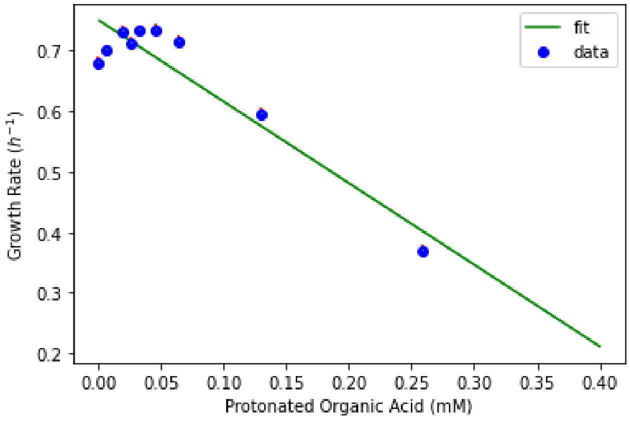

</td></tr>
<tr><td>

D3.

Ghose and Tyagi

$$\mu = \mu_m \left(1 - \frac{L}{L^*}\right)\left(\frac{G}{K_G + G}\right)$$

$$L^* = 0.916\ mM$$
$$R^2 = 0.40$$

</td><td>

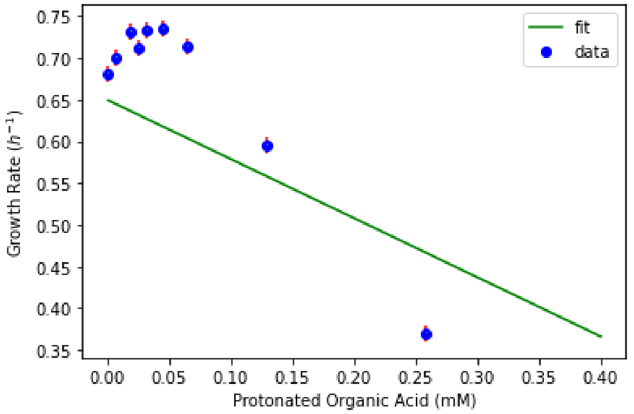

</td></tr>
</table>

**D4.** Aiba and co-workers<br>$$\mu = \mu_m \left(\frac{G}{K_G + G}\right) e^{-KL}$$<br>$K = 1.11\ mM^{-1}$<br>$R^2 = 0.36$	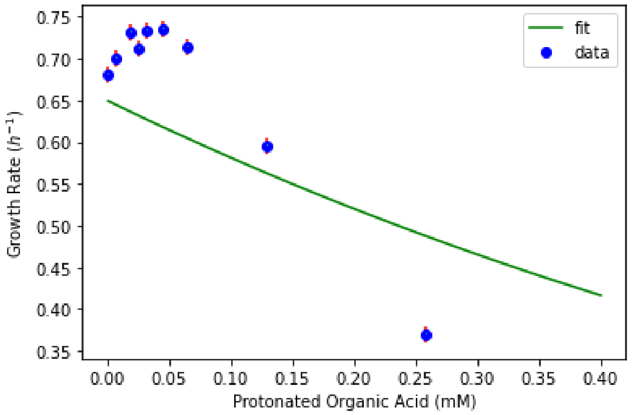
**D5.** Jerusalimsky and Neronova<br>$$\mu = \mu_m \left(\frac{G}{K_G + G}\right)\left(\frac{K_I}{K_I + L}\right)$$<br>$K_I = 0.894\ mM$<br>$R^2 = 0.33$	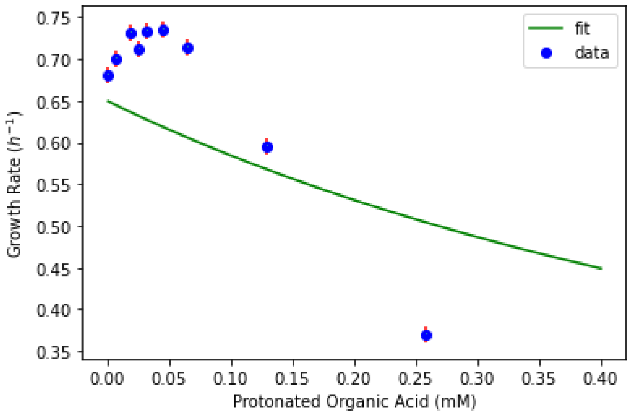
**D6.** Aiba and co-workers dual substrate derivative (single μ)<br>$$\mu = \mu_m \left[\left(\frac{G}{K_G + G}\right) + \left(\frac{L}{K_L + L}\right)\right] e^{-\alpha L}$$<br>$K_L = 0.0743\ mM$<br>$\alpha = 4.44\ mM^{-1}$<br>$R^2 = 0.98$	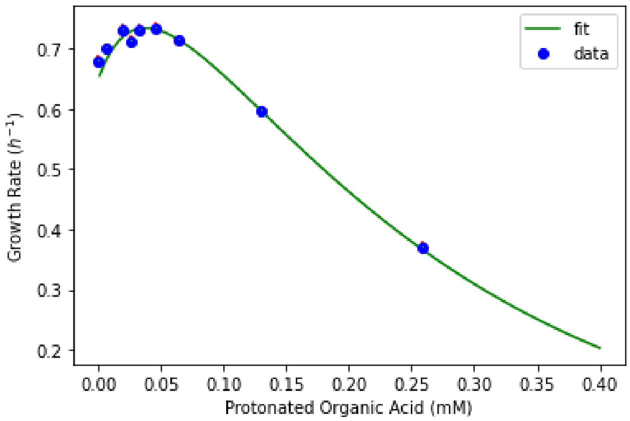

**D7.** Classic model dual substrate derivative (single μ) $\mu = \mu_c\left[\left(\frac{G}{K_G + G}\right) + \left(\frac{L}{K_L + L}\right)\right]\left(\frac{K_I}{K_I + L}\right)$ $K_L = 0.0459\ mM$ $K_I = 0.132\ mM$ $R^2 = 0.96$	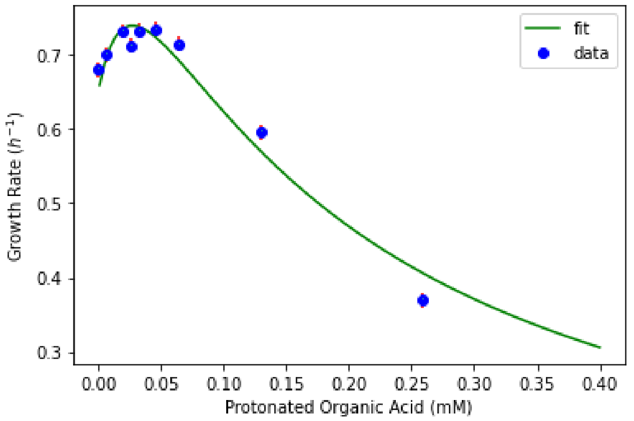
