# Supplementary figures and images for: Environment Constrains Fitness Advantages of Division of Labor in Microbial Consortia Engineered for Metabolite Push or Pull Interactions
Source: mSystems. 2022 Jun 28;7(4):e00051-22. doi: 10.1128/msystems.00051-22 (PMC9426560; doi:10.1128/msystems.00051-22)

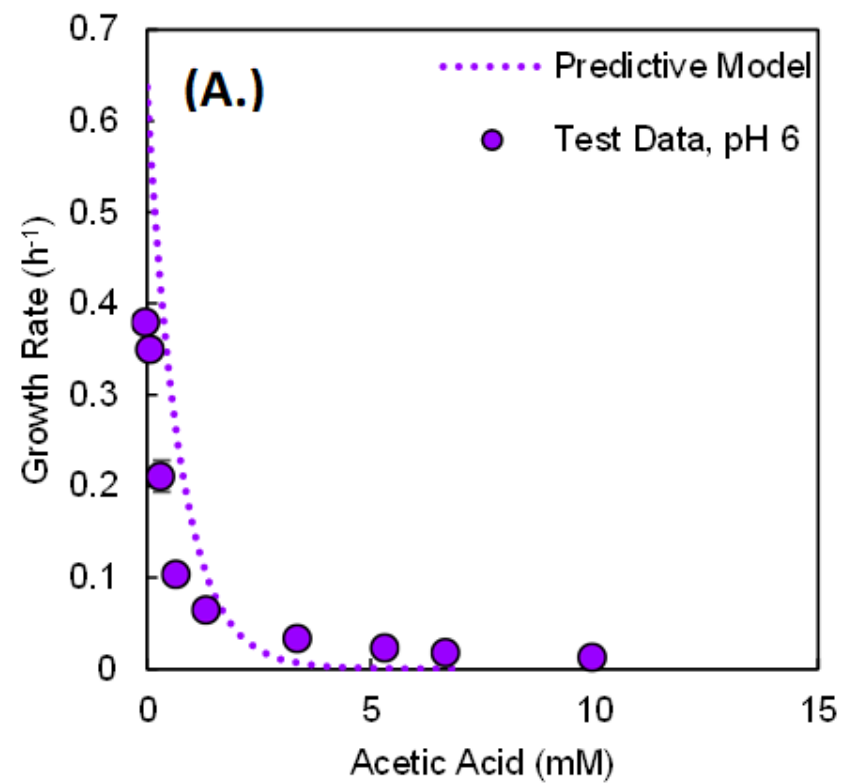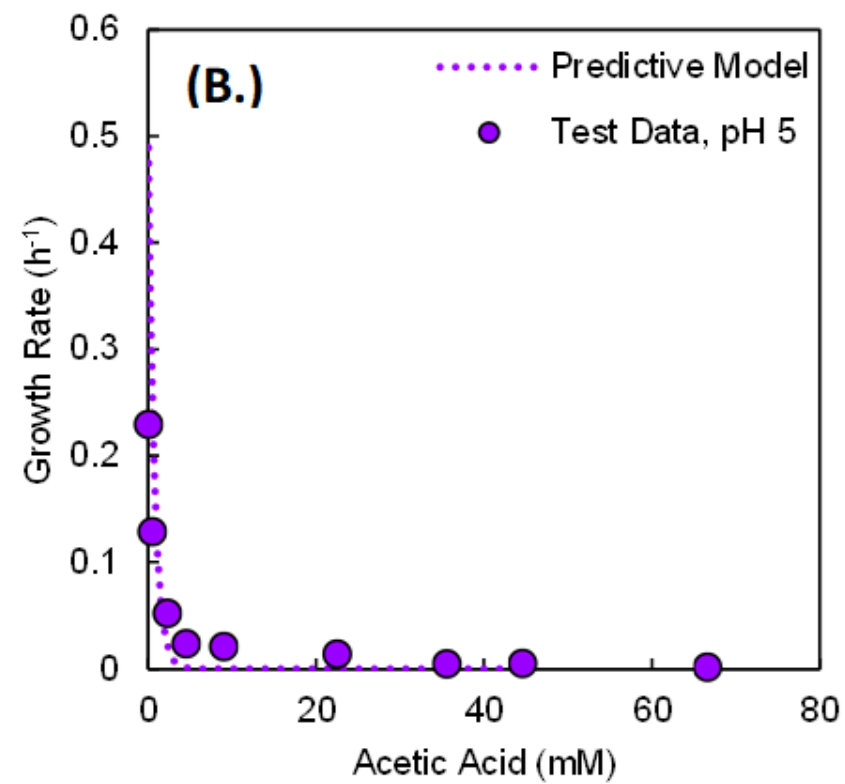

Figure S2.

Supplement: FIG S2 [file msystems.00051-22-s0001.pdf]

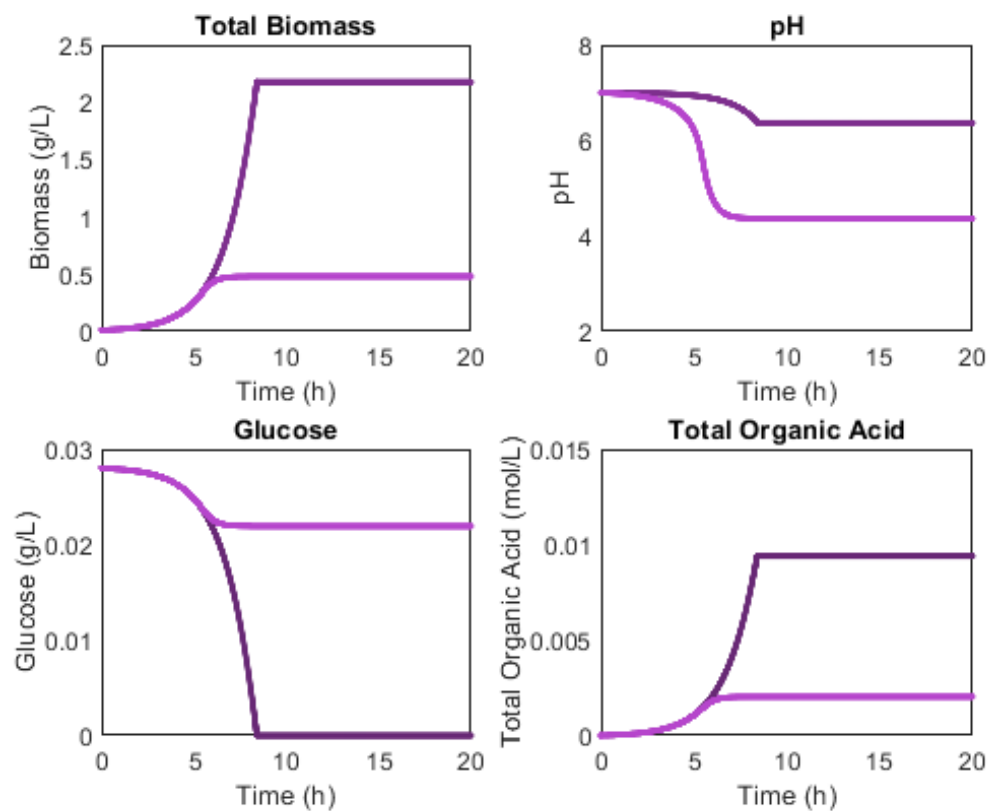

Figure S3A.

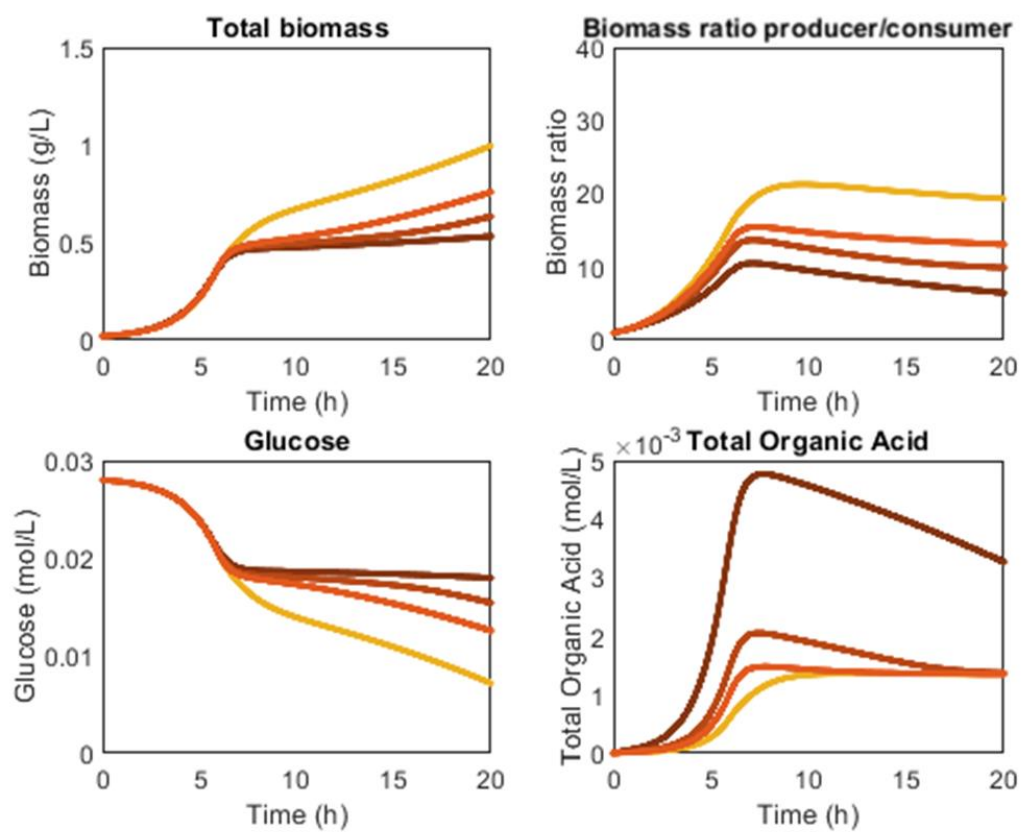

Figure S3B.

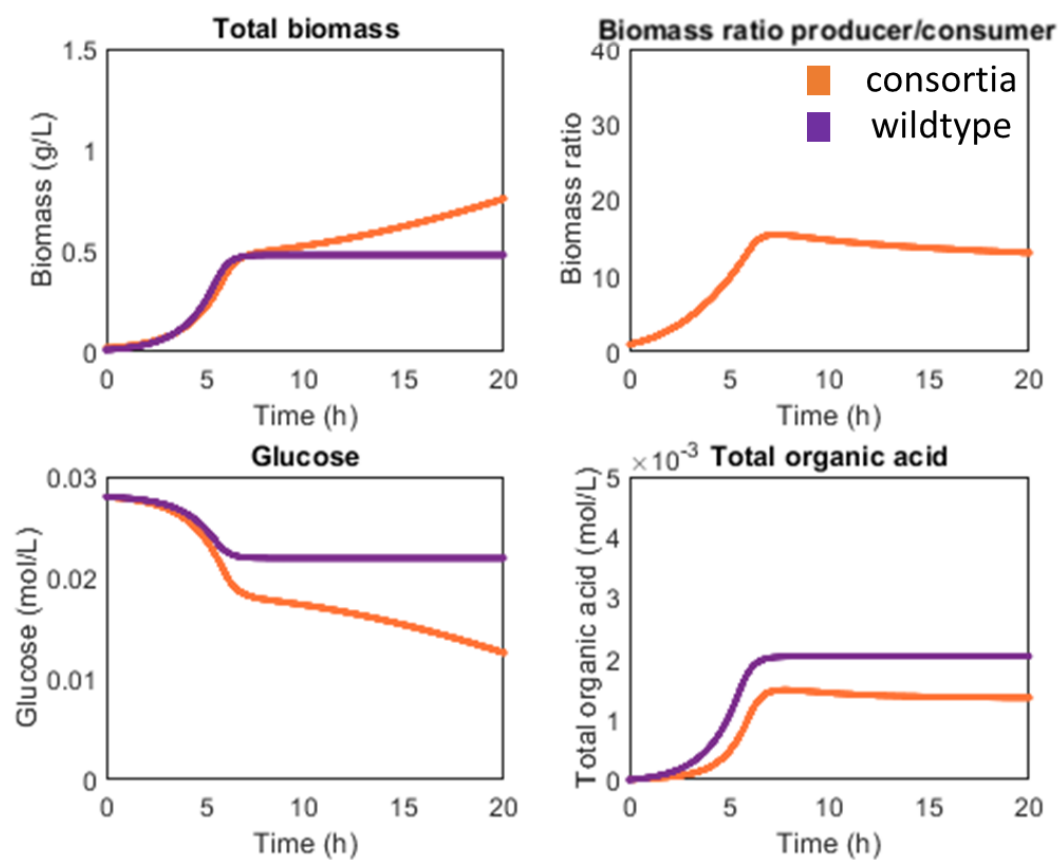

Figure S3C.

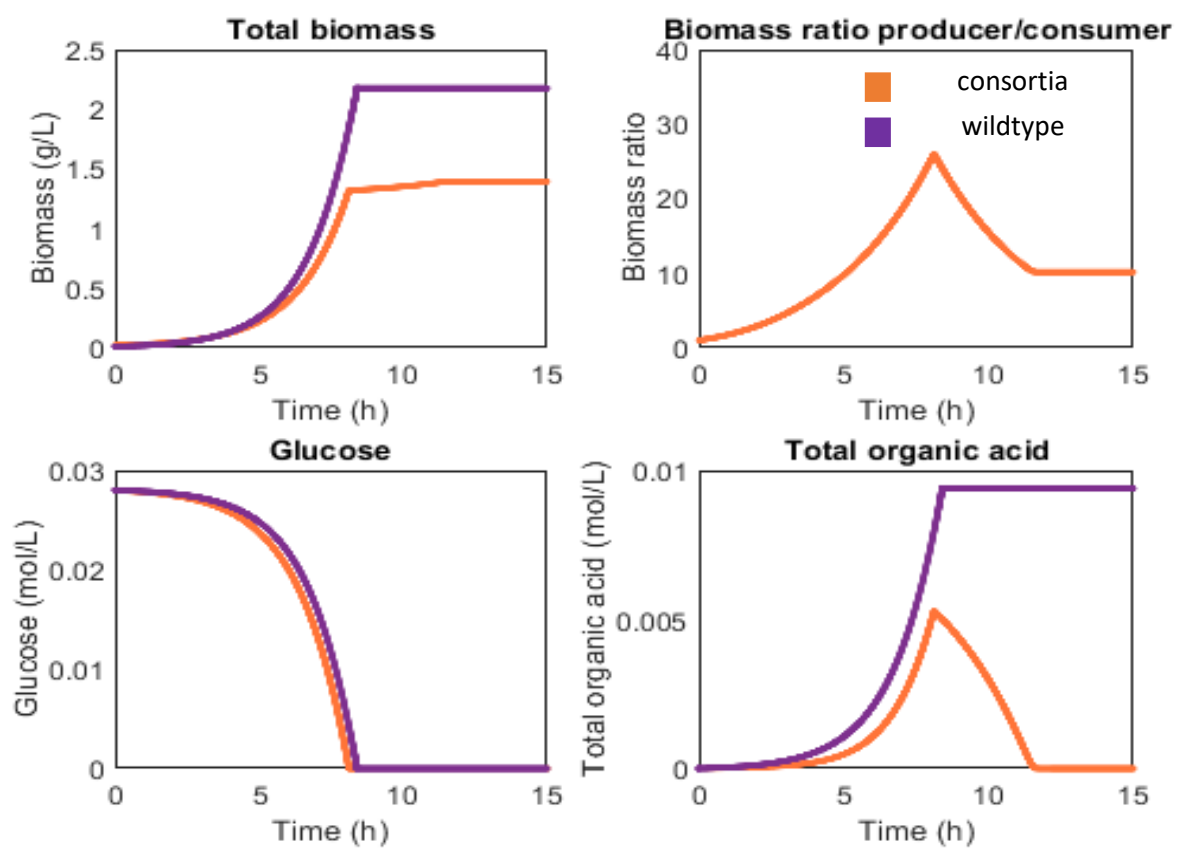

Figure S3D.

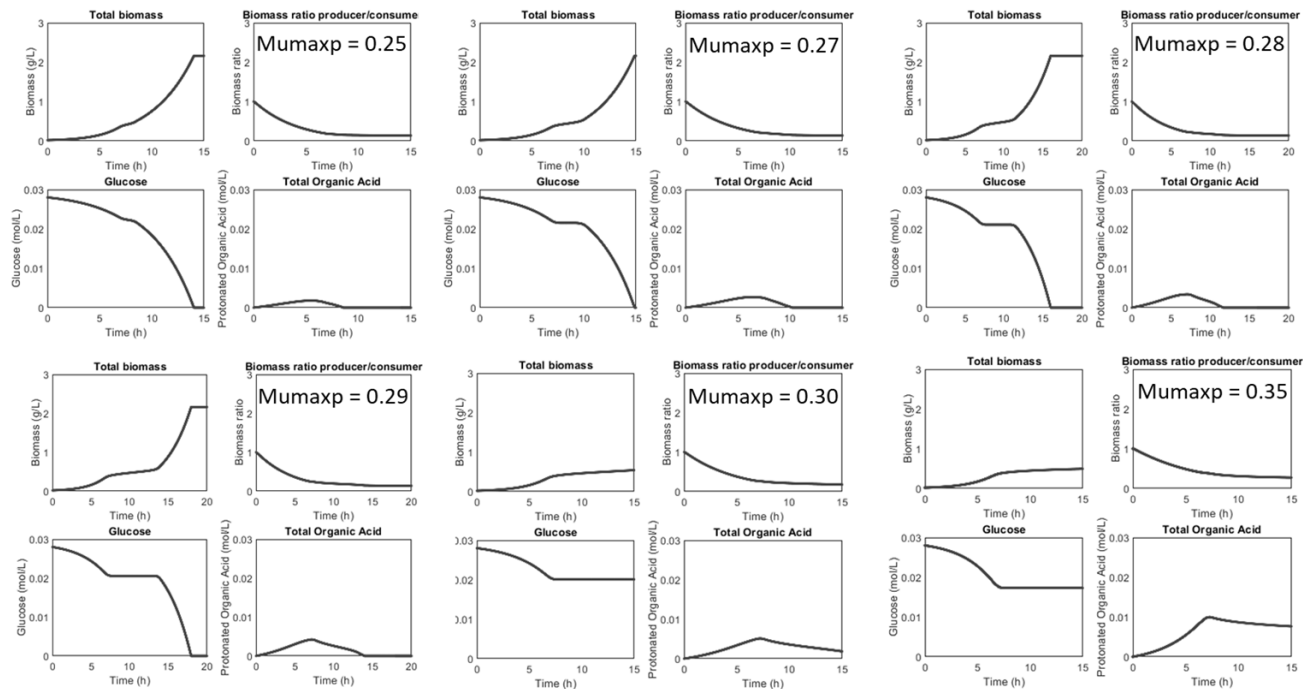

Figure S3E.

Supplement: FIG S3 [file msystems.00051-22-s0006.pdf]
